# Evaluation of *SLC11A1 *as an inflammatory bowel disease candidate gene

**DOI:** 10.1186/1471-2350-6-10

**Published:** 2005-03-09

**Authors:** Nigel PS Crawford, Maurice R Eichenberger, Daniel W Colliver, Robert K Lewis, Gary A Cobbs, Robert E Petras, Susan Galandiuk

**Affiliations:** 1 Digestive Surgery Research Laboratory, Price Institute for Surgical Research, Department of Surgery, University of Louisville School of Medicine, Louisville, KY 40292, USA; 2 Department of Biology, University of Louisville, Louisville, KY 40292, USA; 3 Ameripath Institute of Gastrointestinal Pathology and Digestive Disease, Oakwood Village, OH 44146, USA

## Abstract

**Background:**

Significant evidence suggests that a promoter polymorphism withinthe gene *SLC11A1 *is involved in susceptibility to both autoimmune and infectious disorders. The aim of this study was to evaluate whether SLC11A1 has a role in the susceptibility to inflammatory bowel disease (IBD) by characterizing a promoter polymorphism within the gene and two short tandem repeat (STR) markers in genetic proximity to *SLC11A1*.

**Methods:**

The studied population consisted of 484 Caucasians with IBD, 144 population controls, and 348 non-IBD-affected first-degree relatives of IBD patients. IBD subjects were re-categorized at the sub-disease phenotypic level to characterize possible *SLC11A1 *genotype-phenotype correlations. Polymorphic markers were amplified from germline DNA and typed using gel electrophoresis. Genotype-phenotype correlations were defined using case-control, haplotype, and family-based association studies.

**Results:**

This study did not provide compelling evidence for *SLC11A1 *disease association; most significantly, there was no apparent evidence of *SLC11A1 *promoter allele association in the studied Crohn's disease population.

**Conclusion:**

Our results therefore refute previous studies that have shown *SLC11A1 *promoter polymorphisms are involved in susceptibility to this form of IBD.

## Background

Inflammatory bowel disease (IBD) encompasses a number of disorders that are characterized by chronic inflammation of the gastrointestinal tract. The three specific forms of the disease known as Crohn disease (CD), ulcerative colitis (UC), or the less well-characterized indeterminate colitis (IC), are diagnosed based on the results of clinical observations, histology, radiology, and to a lesser extent, serology. Prominent clinical features include profuse diarrhea, abdominal pain, and increased colorectal cancer risk. IBD affects an estimated one million Americans [1], with UC being the more prevalent form. Current pathogenic models state that IBD develops in a genetically susceptible individual in response to environmental stimuli [2].

The aim of this study was to investigate the role of the gene *SLC11A1 *in the susceptibility to IBD. SLC11A1 is a proton-coupled bivalent metal antiporter that is crucial in early macrophage activation [3]. *SLC11A1 *encodes a highly hydrophobic 550 amino acid membrane protein and is located on the long arm of chromosome 2 (2q35), a region that has not been identified as being within an IBD susceptibility locus. Genetic studies have shown that different alleles of a (GT)_n _dinucleotide repeat polymorphism located within the promoter region of *SLC11A1 *cause susceptibility to different human diseases. Allele 2 of this polymorphism is associated with susceptibility to intracellular pathogen infection [4], and allele 3 is associated with autoimmune disease susceptibility [3].

SLC11A1 has been implicated in susceptibility to IBD. Previous work from this laboratory [5] has shown that two tetranucleotide short tandem repeat (STR) polymorphisms genetically linked to *SLC11A1 *are associated with CD [5]. The (GT)_n _promoter polymorphism, however, was not examined in that study. A recent study by Kojima et al [6] indicated that promoter polymorphism allele 7 was associated with IBD in a Japanese population.

In our current work, we used a case-control association study to define the role of the (GT)_n _promoter polymorphism in susceptibility to IBD and to further characterize the STR markers previously described [5]. These markers (D2S434 and D2S1323) are 0.67 Mbp proximal and 3.44 Mbp distal of *SLC11A1*, respectively.

## Methods

### Population

This prospective study was approved by the University of Louisville Institutional Review Board. Written informed consent was obtained from all subjects. Patients were derived from a University-based colon and rectal surgery practice. Initial IBD diagnoses were determined through radiological, endoscopic, and/or histopathological studies. Histology was available in all cases.

The study population consisted of a total of 628 Caucasians, including 254 unrelated individuals with CD (63% women), 165 with UC (53% women), 65 with IC (70% women), and 144 population controls (74% women). Demographic data for the IBD population is shown in Table 1. For the purposes of more accurate phenotyping, the CD group was subdivided based on the Vienna classification [7]. This categorizes CD patients based on age of onset (A), location of disease (L), and disease behavior (B). With regard to age of onset, 175 of 254 (69%) CD patients were diagnosed at < 40 years of age (A1 group) and 47 of 254 (18%) had disease diagnosed at ≥40 years of age (A2 group). Information regarding age of onset was not available for 32 of 254 patients (13%). Regarding disease location, 70 of 254 (28%) had terminal ileal disease (L1 group), 152 of 254 (60%) had purely colonic and/or ileocolic (ileal and colonic) disease (L2/L3 groups), and 32 of 254 (13%) had disease located proximal to terminal ileum (L4 group). The L2 and L3 groups were combined for the purposes of this study, since colonic disease constitutes the primary focus of our group. Regarding disease behavior, 96 of 254 (38%) had uncomplicated inflammatory disease (B1 group), 28 of 254 (11%) had stricturing disease (B2 group), and 130 of 254 (51%) had penetrating disease (B3 group).

A total of 348 non-affected first-degree relatives of IBD patients were available for the study. There were 63 CD families (29 triads, 30 discordant sibling pairs, 4 triad/discordant sibling pair combinations), 43 UC families (11 triads, 24 discordant sibling pairs, 8 triad/discordant sibling pair combinations), and 14 IC families (5 triads, 5 discordant sibling pairs, 4 triad/discordant sibling pair combinations). In addition, there were 17 CD families, 18 UC families, and 9 IC families who could not be classified as either a triad or discordant sibling pairs.

### Markers and genotyping

STRs were amplified from genomic DNA extracted from peripheral leukocytes. Polymerase chain reaction (PCR) primers were custom-synthesized (Proligo, La Jolla, CA). PCR amplification of the *SLC11A1 *promoter polymorphism was performed using 100 ng template DNA, 2.5 mM MgCl_2_, 50 mM KCl, 10 mM of tris/HCl, 0.2 μM of each dNTP, 200 pmol of each primer, and 1 unit of Taq polymerase (Promega, Madison, WI). Primer sequences were as follows: 5'GACATGAAGACTCGCATTAG3' & 5'TCAAGTCTCCACCAGCCTAGT3'. The product was amplified using the following conditions: 94°C for 10 min, followed by 25 cycles of 94°C for 30 sec, 55°C for 75 sec, and 72°C for 20 sec. A final extension step was run at 72°C for 6 min. Genotyping was performed using gel electrophoresis (Spreadex EL 300 S-100 gel [Elchrom Scientific, Lake Park, FL]). PCR amplification of the STR markers D2S434 and D2S1323 was performed as described previously [5].

### Statistical analyses

#### Case-control tests of association

Case-control analyses were performed using STR genotype-allele frequencies in cohorts subdivided on the variables outlined in Table 1. Correction for multiple testing was performed by using Storey's q-value method, where p-values were adjusted according to the experimental false discovery rate (FDR) [8]. The global null hypothesis of no difference in genotype-allele frequency between controls and any of the other groups was tested by using Fisher's method [9], which was used to combine all of the control group comparison global p-values obtained for each of the three markers. Q-values were calculated from these global p-values using the QVALUE program [10] with the tuning parameter λ = 0, which dictates the assumption π_o _= 1, where π_o _is the proportion of tests in which the null hypothesis is true.

To limit the number of individual disease-control genotype-allele comparisons, further analyses were only performed in those disease groups possessing a global test q-value <0.05. Therefore, 2 × 2 contingency tables were only constructed for genotypes with a global test q-value meeting this condition. Disease *versus *control comparisons were performed using Fisher's exact test and corrected for multiple testing using the q-value method as described above.

Since IBD affecting the colon is the primary focus of our research, the L2 colonic CD and L3 ileocolonic CD groups were combined, enabling maximization of statistical power. We argue that this approach will not impair the validity of statistical analyses given that the Vienna Classification is somewhat arbitrary and that group genetic homogeneity may be increased by combining all those cases with colonic IBD. Furthermore, owing to the relatively low numbers of individuals with CD proximal to the terminal ileum (L4 group, n = 32), this disease subgroup was excluded from case-control analyses.

#### Family-based tests of association

Family-based tests used included the pedigree disequilibrium test (PDT) [11,12] and a likelihood ratio test implemented in the computer program TRANSMIT [13]. PDT analysis has distinct advantages over other family-based tests of association. PDT allows for analysis of data from related nuclear families and discordant sibling pairs from extended pedigrees. The likelihood ratio test, as implemented in the computer program TRANSMIT [13], can account for missing parental genetic data by inferring parental genotypes based on the genotypes of their offspring. To maximize power, family-based analyses were not performed at the sub-phenotypic level.

## Results

### Allele and genotype frequencies

Alleles for the *SLC11A1 *promoter polymorphism STR D2S434 and STR D2S1323 were named numerically according to molecular weight, with "1" representing a larger allele than "2" and so on. Allele distributions are shown in Table 2.

DNA sequence analysis of the three *SLC11A1 *promoter polymorphism alleles identified in the study population revealed the following sequences:

Allele 1: T(GT)_5_AC(GT)_5_AC(GT)_11_GGCAGA(G)_6_

Allele 2: T(GT)_5_AC(GT)_5_AC(GT)_10_GGCAGA(G)_6_

Allele 3: T(GT)_5_AC(GT)_5_AC(GT)_9_GGCAGA(G)_6_

These sequences corresponded to alleles 1, 2, and 3 as described by Searle and Blackwell [14]. Alleles 4–7 were not identified in this population.

### Case-control association studies

Results from global disease *versus *control comparisons of the studied markers are shown in Table 3. Analysis of genotype and allele frequencies for the (GT)_n _promoter polymorphism did not reveal any evidence of association. Additionally, we studied STR markers in genetic proximity to *SLC11A1*. The STR D2S1323 exhibited evidence of association with the test of the global null hypothesis of homogeneity of all groups exhibiting statistical significance for allele frequencies (q = 0.050, p = 0.008). To limit multiple testing, individual pair-wise disease *versus *control comparisons were only performed for D2S1323 alleles. These analyses showed over-representation of allele 1 in the UC group (q = 0.022, p = 0.005; UC frequency 78 of 100 alleles [78%] *versus *control frequency 54 of 92 alleles [59%]). The STR D2S434 did not reveal any evidence of association. Further analyses of genotype and allele data for all markers did not show a correlation with either age of onset or disease behavior.

### Family-based association studies

Family-based tests were performed for each of the three markers. Neither the likelihood ratio test nor the PDT revealed significant evidence of association for any of these markers.

## Discussion

No association was observed between any *SLC11A1 *promoter polymorphism alleles and any IBD sub-phenotypic group in this population, which unexpectedly refutes the significant evidence of others [6]. This result was somewhat surprising, given that many studies have shown *SLC11A1 *promoter polymorphisms to be involved in susceptibility to autoimmune disorders and disorders that are characterized by a high degree of immunological dysregulation, including IBD [3]. Searle and Blackwell [14] investigated the enhancer properties of each of the four identified promoter polymorphism alleles. Allele 3 was found to have endogenous enhancer activity, whereas alleles 1, 2, and 4 were poor promoters in the absence of exogenous stimuli. Stimulation of macrophages with bacterial lipopolysaccharide (LPS) had minimal effects on *SLC11A1 *expression driven by alleles 1, 2, and 4; however, allele 3-driven *SLC11A1 *expression was enhanced. It has long been recognized that altered expression of *SLC11A1 *in response to LPS is a key event in the activation of macrophages upon contact with a variety of pathogens [15].

Based on those observations, we hypothesized that over-representation of promoter allele 3 in CD patients could lead to hyperactivation of bowel wall macrophages that are chronically exposed to high levels of LPS. This could subsequently cause the autoimmune-like phenotype characteristic of IBD. This hypothesis must be rejected in this cohort due to the absence of association with promoter allele 3 in any IBD subgroup. Analysis of intragenic single nucleotide polymorphisms are required to conclusively exclude *SLC11A1 *as an IBD candidate gene.

D2S1323 was associated with UC in this population. Indeed, this was the only evidence for association in any of the studied STR markers in proximity to *SLC11A1*. This association was not, however, confirmed upon family-based statistical analysis. The current finding was particularly surprising to us, given that our previous study showed this STR to be associated with CD rather than UC [5]. The significance of this finding is thus uncertain, and the divergent outcomes from the current and earlier studies could be considered somewhat concerning. We do, however, believe that a number of prominent features of the current study render our results of higher validity than those of our previous study. An important feature of our current work is that all histological specimens from our subjects have been reviewed by a single gastrointestinal pathologist. This was not the case in the earlier study, and diagnostic misclassification (i.e., UC and/or IC incorrectly diagnosed as CD and *vice versa*) may have been a confounding variable that may have in part led to what we now consider the false-positive association observed in the CD population. It should also be noted that the current study contains over two-fold the number of subjects as compared with the previous work, and thus statistical power has been significantly increased. Furthermore, our current statistical methodology is now far superior, and we argue that our stringent means of correcting for the effects of multiple testing effectively minimizes the likelihood of detecting a false-positive association. This is a vast improvement over our earlier study in which compounding of a type 1 error was not taken into account. Finally, genotyping in the earlier study was performed on specimens with a rather heterogeneous nature and consisted of genetic data derived from DNA extracted from both surgical specimens (i.e., somatic DNA) and leukocytes (i.e., germline DNA). In our current study, genetic material has only been isolated from leukocytes, thus reducing the possibility of detecting variations within somatic DNA.

## Conclusion

In summary, we have characterized the *SLC11A1 *(GT)_n _promoter polymorphism in IBD sub-phenotypic groups and accept the null hypothesis as confirmed by family-based testing. We did, however, identify an association between an allele of the STR D2S1323 and UC upon case-control analysis. This association was not confirmed on family-based and haplotype testing, and its actual significance in IBD remains to be seen.

## Competing interests

The author(s) declare that they have no competing interests.

## Authors' contributions

NPSC performed genotyping, PDT analyses, and drafted the manuscript; MRE performed genotyping and was responsible for coordination of laboratory work; authors DWC and RKL performed genotyping; GAC performed statistical analyses; REP performed re-review of colonic IBD histology; SG collected all patient samples, participated in study design, coordination, and manuscript preparation and obtained funding for this work. All authors read and approved the final manuscript.

## Pre-publication history

The pre-publication history for this paper can be accessed here:



## Figures and Tables

**Table 1:** General characteristics of the studied inflammatory bowel disease (IBD) population.

**Disease characteristic**	**Crohn's disease (n = 254)**	**Ulcerative Colitis (n = 165)**	**Indeterminate colitis (n = 65)**
Average length of clinical follow-up (range, yrs)	4.6 (0–16)	4.3 (0–14)	5.6 (0–12)
Age of IBD onset (range, yrs)	29.2 (6–81)	34.4 (10–75)	29.7 (12–64)
Surgical treatment for IBD complications (% received)	75%	67%	82%
First-degree relative with IBD	36%	20%	22%
Extraintestinal manifestations^(a)^	43%	36%	49%

**(a) **Extraintestinal manifestations are defined as IBD-related disease outside the gastrointestinal tract involving joints, eyes, hepatobiliary system, or the skin.

**Table 2:** Polymorphism allele frequencies.

***SLC11A1 *Promoter Allele**	**Ulcerative Colitis**		**Indeterminate Colitis**		**Ileocolic Crohn's Disease (L1)**		**Colonic Crohn's Disease (L2/3)**		**Controls**	
	
	**N**	**%**	**N**	**%**	**N**	**%**	**N**	**%**	**N**	**%**
1	0	0	0	0	0	0	0	0	2	1
2	40	21	19	21	28	29	44	25	42	23
3	152	79	71	79	68	71	132	75	136	76
Total	192		90		96		176		180	

										
**D2S434 Allele**	**N**	**%**	**N**	**%**	**N**	**%**	**N**	**%**	**N**	**%**

3	23	30	22	24	22	25	48	12	25	14
4	53	29	23	26	23	26	46	27	52	30
5	43	30	23	27	24	26	48	22	47	27
6	23	19	17	9	8	19	30	12	30	17
Pooled^(a)^	16	14	5	12	11	6	22	8	20	11
Total	158		90		88		194		174	

										
**D2S1323 Allele**	**N**	**%**	**N**	**%**	**N**	**%**	**N**	**%**	**N**	**%**

1	78	78	28	70	39	72	83	67	54	59
2	22	22	12	30	15	28	41	33	38	41
Total	100		40		54		124		92	

**(a) **Pooled alleles with frequency <5%

**Table 3:** Case-control association global q-value and p-values for disease group v*ersus *control comparisons.

**STR marker**		**Disease *versus *control comparison global p-value**				**Global test of homogeneity^(a)^**	
		
		**Ulcerative colitis**	**Indeterminate colitis**	**Terminal ileal Crohn's disease (L1)**	**Colonic Crohn's disease (L2/3)**	**Combined p-value**	**q-value**
Promoter (GT)_n_	Genotype	0.553	0.424	0.743	0.768	0.855	0.855
	Allele	0.457	0.548	0.473	1.000	0.832	0.855
							
D2S434	Genotype	0.390	0.345	0.365	0.705	0.562	0.842
	Allele	0.930	0.191	0.157	0.153	0.206	0.412
							
D2S1323	Genotype	0.027	0.424	0.202	0.490	0.093	0.279
	Allele	0.005	0.247	0.111	0.253	**0.008**	**0.050**

**(a) **Statistically significant comparisons following correction for multiple testing are shown in bold typeface
